# Comparing Dosimetry of Heart and Left Anterior Descending Artery Exposure in Carcinoma Esophagus Patients: Volumetric Arc Therapy Versus Intensity-Modulated Radiotherapy

**DOI:** 10.7759/cureus.68182

**Published:** 2024-08-30

**Authors:** Vishwadeep Mishra, Shwetima Chaudhary, Prarabdh Singh, Laxman Pandey, Archana Pandey, Rachita Chatterjee

**Affiliations:** 1 Radiation Oncology, All India Institute of Medical Sciences, Gorakhpur, Gorakhpur, IND; 2 Radiation Oncology, T.S Misra Medical College and Hospital, Lucknow, IND; 3 Radiation Oncology, Homi Bhabha Cancer Hospital and Mahamana Pandit Madan Mohan Malaviya Cancer Centre, Varanasi, Varanasi, IND; 4 Radiation Oncology, Rohilkhand Medical College and Hospital, Bareilly, IND; 5 Pediatric Medicine, Baba Raghav Das (BRD) Medical College, Gorakhpur, IND

**Keywords:** oesophagus cancer, radiation therapy, dosimetric analysis, cardiac sparing, imrt, vmat

## Abstract

Introduction

Esophageal cancer remains a leading cause of cancer-related mortality worldwide, with chemoradiotherapy being a cornerstone of its treatment. Ensuring precise radiation delivery is critical, as it minimizes exposure to surrounding healthy tissues, particularly vital structures like the heart and the left anterior descending artery (LAD). Volumetric arc therapy (VMAT) and intensity-modulated radiotherapy (IMRT) are two advanced radiotherapy techniques that offer enhanced dose conformity and reduced toxicity. This study conducts a retrospective dosimetric analysis to compare the effectiveness of VMAT and IMRT in sparing cardiac substructures and the LAD in patients with carcinoma of the esophagus.

Methods

Ten patients with middle-third esophageal cancer were treated using the VMAT technique with two coplanar arcs. These patients were retrospectively re-planned with IMRT using 7-9 fields on the Varian TrueBeam linear accelerator between June 2023 and December 2023. VMAT planning involved a two-phase approach: 45 Gy in 25 fractions followed by a boost of 5.4 Gy in three fractions. Dose-volume histograms were analyzed and compared for the planning target volume (PTV), heart and its substructures (including the right atrium, right ventricle, left atrium, and left ventricle), and the LAD. Statistical significance was determined using paired t-tests with a significance level set at P < 0.05.

Results

PTV coverage was comparable between VMAT and IMRT. VMAT resulted in higher low-dose exposure (V5 and V10) but offered better sparing at moderate doses (V20 and V40) for the heart. The LAD benefited from reduced high-dose exposure with VMAT. For other cardiac substructures, VMAT generally showed higher low-dose exposure but provided superior sparing at moderate doses compared to IMRT.

Conclusions

VMAT offers notable dosimetric advantages in sparing critical cardiac structures compared to IMRT for treating patients with middle third esophageal cancer. Long-term follow-up studies are needed to assess how these dosimetric benefits influence coronary artery disease and other cardiac complications.

## Introduction

Esophageal cancer is becoming the 11th most common cancer worldwide and the leading cause of cancer-related deaths [1]. Concurrent chemoradiotherapy with or without surgery has become a standard treatment for patients with locally advanced esophageal cancer [2,3]. Volumetric arc therapy (VMAT) can help reduce side effects by targeting the cancer more precisely and sparing healthy tissue [3]. Treating other cancers like breast cancer and Hodgkin’s disease radiation can increase the risk of heart problems for which factors have been identified, including the heart volume, the total amount of radiation, and the fraction size [4-9]. The treatment of carcinoma esophagus generally results in higher cardiac doses compared to treatments for breast cancer or Hodgkin’s lymphoma because of the esophagus’s close proximity to the heart and the necessity for high radiation doses [10]. Due to the close proximity of the esophagus to the heart, cardiac toxicity is a major concern in radiation treatment (RT) of esophageal cancer. The Radiation Therapy Oncology Group (RTOG) 0617 trial also demonstrated that lowering the radiation dose to the heart was linked to better overall survival (OS) [11]. Previously, the entire heart was considered an organ at risk. Recent studies have demonstrated that dose to cardiac substructures like heart chambers and vessels may better predict cardiac toxicities. The cardiovascular consequences stemming from radiation exposure typically exhibit a prolonged latent period, often presenting as subclinical changes such as coronary artery disease (CAD), ischemia, myocardial fibrosis, and valvular insufficiency alongside pericardial disease [12,13]. Limited data exist regarding cardiac complications arising from radiation therapy (RT) for esophagus cancer, largely attributed to the typically poor long-term survival rates of these patients. Existing literature offers conflicting findings regarding the correlation between radiation dose to the heart and associated cardiac toxicity [13].

As far as we are aware, there are presently no definitive guidelines or published studies addressing the prognosis of the cardiovascular system following the treatment of esophageal cancer, so we conducted this retrospective dosimetric analysis for various cardiac vascular structures to see dosimetric parameters among esophageal cancer patients undergoing VMAT and intensity-modulated radiotherapy (IMRT) planning.

## Materials and methods

This retrospective observational study analyzed 10 patients at Rohilkhand Medical College and Hospital, Bareilly, India, with middle-third esophageal cancer, all of whom were treated with the VMAT technique between July 2023 and December 2023. The patients’ ages ranged between 50 and 70 years, with an average age of 66.5 years. The histology of the tumors in most patients was moderately differentiated squamous cell carcinoma (6 out of 10), followed by well-differentiated squamous cell carcinoma. These patients were selected to evaluate the dosimetric differences between VMAT and IMRT. For each patient, a theoretical IMRT treatment plan was generated, resulting in 10 VMAT (Group I) and 10 IMRT plans (Group II) for comparison. The research was approved by the Institutional Ethics Committee of Rohilkhand Medical College and Hospital (approval number IEC/RMCH/03/2023/May).

Inclusion and exclusion criteria

Patients who had middle-third esophageal cancer and were aged ≥18 years and ≤70 years with squamous cell carcinoma were included in this study. Patients who had undergone previous surgery or radiotherapy and presented with other metastatic diseases were excluded.

Radiotherapy planning

Immobilization and Simulation

A thoracic mold was made using a uniform thermoplastic cast in the supine position with the hand kept above the head. IV and oral contrast-enhanced CT simulation imaging with a slice thickness of 2.5 mm was taken from C2 to L4 vertebrae.

Delineation of PTV and Organs at Risk (OARs)

The gross tumor volume (GTV), clinical target volume (CTV), planning target volume (PTV), and lymph nodes, as well as the OARs including lung, heart, and spinal cord, were contoured according to the RTOG 0436 protocol. The GTV was contoured based on visible tumors or lymph nodes on CT scans with fusion with PET CT in most of the patients. The CTV was delineated with 3 cm superior-inferior margins and 1.5 cm radial margin with respect to the GTV. For the lymph nodes, 1 cm uniform margins were given in all directions. Anatomical boundaries like lungs and bones were considered while contouring CTV. The PTV was delineated with an additional 0.5 cm margin to the CTV, depending on GTV delineation accuracy and nearby critical structures. The heart was delineated in conjunction with the pericardial sac, starting from its base, which was identified by the inferior aspect of the pulmonary artery traversing the midline and extending inferiorly to the apex. The left ventricle and left anterior descending arteries were outlined following the guidelines provided by the cardiac contouring atlas. The optimal planning objectives for both techniques were specified as the dose to the PTV between 95% and 107% relative to the 100% prescription point.

VMAT planning

VMAT plans were created using the Varian Eclipse Treatment Planning system (Version 17.00 system). Planning is performed using direct aperture optimization, where we have used two opposite full arcs with 177 points to provide adequate coverage of targets. The calculation algorithm used for calculation was an anisotropic analytical algorithm with a calculation grid size of 2.5 mm.

The virtual IMRT plans were created using the same CT imaging data and target delineation as the original VMAT plans, ensuring consistency across the two treatment modalities. Both plans were optimized according to standard clinical guidelines, focusing on achieving adequate PTV coverage while minimizing exposure to critical structures, particularly the heart and left anterior descending artery (LAD).

IMRT planning

IMRT plans were generated virtually and optimized to cover at least 95% of the PTV with 95% of the prescribed dose while minimizing doses to OARs. Inverse planning optimization was performed using Eclipse TPS. Optimization included 100 iterations followed by a semi-automatic segmentation process. A seven non-coplanar field beam arrangement was employed for all IMRT plans, with gantry angles of 0°, 51°, 102°, 153°, 204°, 255°, and 306°. The isocenter was consistent across all IMRT plans. Similar to VMAT planning, the normal tissue objective feature in optimization was applied to all IMRT plans.

Dose prescription

The dose was prescribed in two phases: Phase 1: 45 Gy in 25 fractions at 1.8 Gy per fraction and Phase II (boost): 5.4 Gy in three fractions at 1.8 Gy per fraction.

Plan evaluation

All treatment plans underwent evaluation using the standard dose-volume histogram. For the PTV, the parameters analyzed included D99%, D95%, maximum dose, and minimum dose. Dosimetry of heart and LAD was analyzed in terms of V5%, V10%, V20%, V40%, V50%, minimum (Dmin), and maximum dose (Dmax). V5 means volume receiving 5Gy and so on for other doses.

Statistical analysis

To determine statistical significance, two-tailed paired t-tests were conducted, with significance set at P-values < 0.05. Statistical analyses were performed using SPSS for Windows, Version 16.0 (Released 2007; SPSS Inc., Chicago, USA).

## Results

Our retrospective study included 10 patients diagnosed with esophageal carcinoma, ranging from stage IB2 to IVA. The primary objective of this comparison was to evaluate and contrast the dosimetric parameters between VMAT and IMRT, specifically looking at dose distributions to the heart, LAD artery, and other cardiac substructures. Six patients were in stage III, and four patients were in stage II as per the American Joint Committee on Cancer (AJCC) 8th edition. The patient’s ages ranged from 50 to 70 years, with an average age of 66.5. Most of the patients presented with dysphagia and/or weight loss. The histology of all the patients was squamous cell carcinoma.

**Figure 1:**
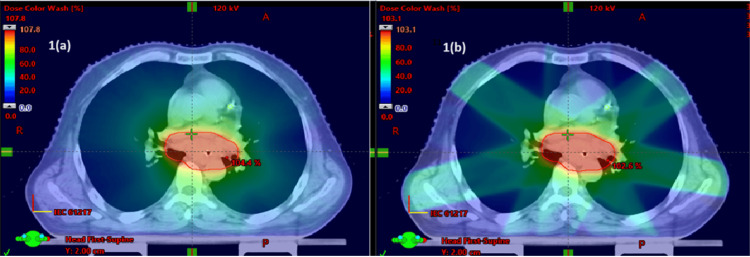
Beam arrangements of (a) VMAT and (b) IMRT IMRT, intensity-modulated radiotherapy; VMAT, volumetric arc therapy

**Figure 2:**
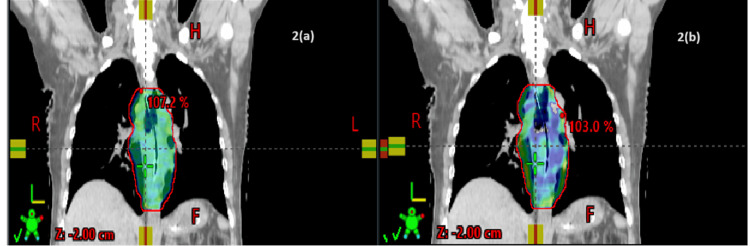
Dose distribution in the coronal section: (a) VMAT and (b) IMRT IMRT, intensity-modulated radiotherapy; VMAT, volumetric arc therapy

**Figure 3:**
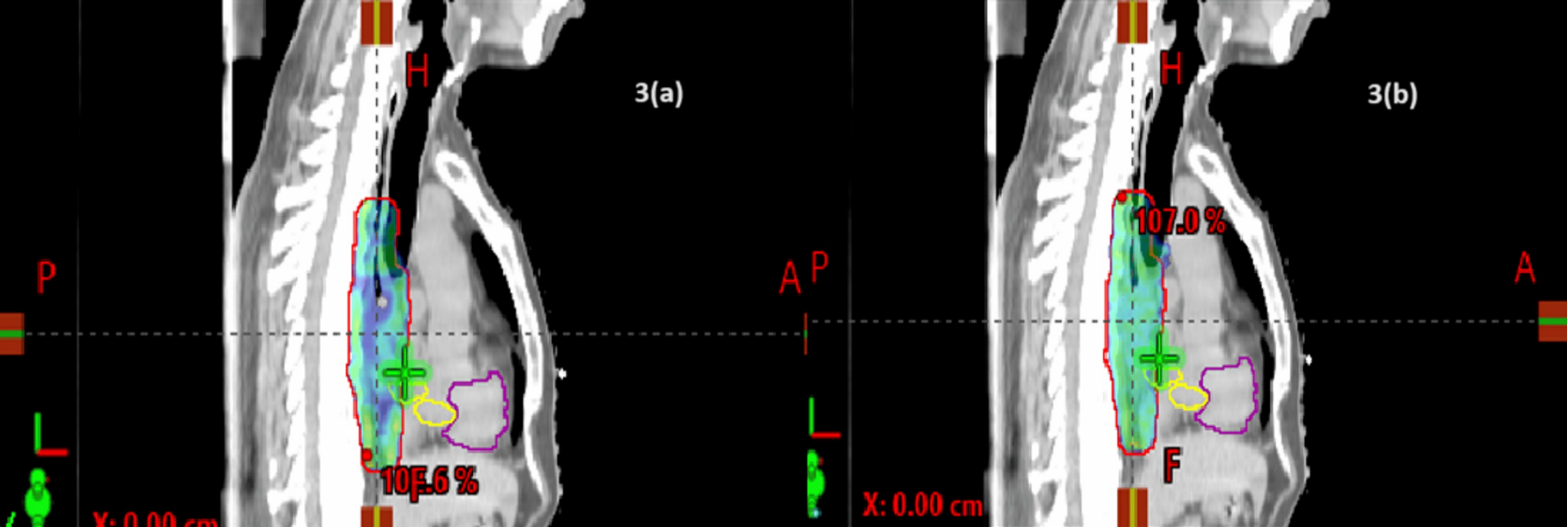
Dose distribution in the sagittal section: (a) VMAT and (b) IMRT IMRT, intensity-modulated radiotherapy; VMAT, volumetric arc therapy

**Figure 4:**
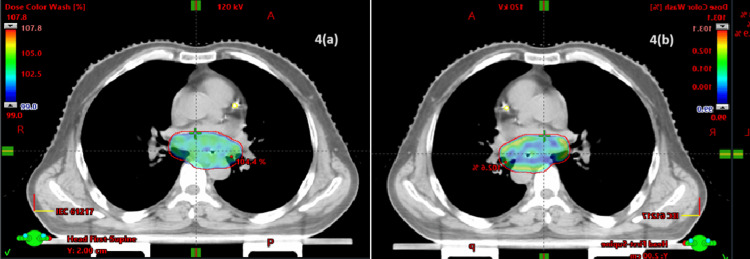
Dose distribution in the axial section: (a) VMAT and (b) IMRT IMRT, intensity-modulated radiotherapy; VMAT, volumetric arc therapy

Dosimetric results showed that the PTV coverage was similar between Group I and Group II (Figures 1-4).

For PTV, the mean dosimetric parameters were D99% (IMRT: 46.39 Gy, VMAT: 45.22 Gy), D95% (IMRT: 48.27 Gy, VMAT: 46.61 Gy), Dmax (IMRT: 52.79 Gy, VMAT: 52.26 Gy), and Dmin (IMRT: 42.55 Gy, VMAT: 42.46 Gy). There were no statistically significant differences between VMAT and IMRT in these parameters (Table 1).

**Table 1:** Mean dosimetric parameters for ensuring PTV coverage in both groups PTV, planning target volume

Dosimetric parameters (PTV)	Group I (Gy)	Group II (Gy)	P-value
D99	45.22	46.39	0.63
D95	46.61	48.27	0.49
Dmax	52.26	52.79	0.14
Dmin	42.46	42.55	0.58

For the heart, VMAT resulted in higher V5 and V10 values compared to IMRT (V5: 93.93% vs. 85.11%, V10: 82.25% vs. 76.56%), indicating greater low-dose exposure. However, VMAT showed lower V20 (34.94% vs. 47.35%) and V40 (8.60% vs. 9.84%) values, demonstrating superior sparing at moderate doses. The V50 was slightly higher with VMAT (3.37% vs. 2.47%), but the difference was minimal. For the left ventricle, VMAT demonstrated better sparing at higher doses with lower V20 (26.79% vs. 37.28%) and V40 (2.78% vs. 3.19%) but had higher V5 (82.16% vs. 88.78%) and V10 (74.40% vs. 60.45%) values, suggesting increased low-dose exposure. VMAT also resulted in significantly higher Dmax for the left ventricle (46.20% vs. 45.04%) (Table 2).

**Table 2:** Mean dosimetric parameters for heart and left ventricle

Organ	Dosimetric parameters	Group I	Group II	P-value
Heart	V5 (%)	93.93	85.11	0.19
	V10 (%)	82.25	76.56	0.47
	V20 (%)	34.94	47.35	0.22
	V40 (%)	8.6	9.84	0.88
	V50 (%)	3.37	2.47	0.27
	Dmin (Gy)	4.19	2.79	0.05
	Dmax (Gy)	51.83	44.79	0.14
Left ventricle	V5 (%)	82.16	88.78	0.55
	V10 (%)	74.4	60.45	0.43
	V20 (%)	26.79	37.28	0.38
	V40 (%)	2.78	3.19	0.75
	V50 (%)	0.49	0.82	0.52
	Dmin (Gy)	5.12	3.94	0.53
	Dmax (Gy)	46.2	45.04	0.00017

Regarding the right atrium, VMAT resulted in slightly higher V10 values (95.67% vs. 84.73%), indicating increased low-dose exposure but showed better sparing at higher doses. For the right ventricle, VMAT showed higher V5 (86.97% vs. 83.87%), V10 (70.92% vs. 54.27%), and V20 (50.14% vs. 23.34%) values, suggesting increased exposure at low to moderate doses but achieved a lower Dmax compared to IMRT (Table 3).

**Table 3:** Mean dosimetric parameters for the right ventricle and left atrium

Organ	Dosimetric parameters	Group I	Group II	P-value
Right ventricle	V5 (%)	86.97	83.87	0.78
	V10 (%)	70.92	54.27	0.35
	V20 (%)	50.14	23.34	0.43
	V40 (%)	1.64	1.25	0.79
	V50 (%)	0.37	0	0.34
	Dmin (Gy)	6.17	5.16	0.63
	Dmax (Gy)	35.12	35.6	0.94
Left atrium	V5 (%)	90.3	99.97	0.34
	V10 (%)	90	99.75	0.35
	V20 (%)	86.13	92.59	0.54
	V40 (%)	34.68	42.25	0.32
	V50 (%)	10.89	7.43	0.25
	Dmin (Gy)	17.77	20.75	0.5
	Dmax (Gy)	51.57	50.31	0.29

VMAT provided lower dosimetric values for the left atrium at all dose levels (V5, V10, V20, and V40) except for V50, where VMAT had a higher value (10.89% vs. 7.43%). For the LAD, VMAT demonstrated higher V5 (92.51% vs. 66.78%) and V10 (53.44% vs. 27.26%) values but significantly lower V20 (2.33% vs. 5.73%) values compared to IMRT, suggesting better sparing of the LAD at higher doses (Table 4).

**Table 4:** Mean dosimetric parameters for the right atrium and LAD LAD, left anterior descending artery

Organ	Dosimetric parameters	Group I	Group II	P-value
Right Atrium	V5 (%)	99.78	99.81	0.88
	V10 (%)	95.67	84.73	0.11
	V20 (%)	37.29	55.11	0.16
	V40 (%)	1.71	3.18	0.37
	V50 (%)	0.33	0.09	0.39
	Dmin (Gy)	9.92	7.98	0.16
	Dmax (Gy)	44.47	44.24	0.95
LAD	V5 (%)	92.51	66.78	0.05
	V10 (%)	53.44	27.26	0.09
	V20 (%)	2.33	5.73	0.21
	V40 (%)	0	0	0
	V50 (%)	0	0	0
	Dmin (Gy)	5.41	4.28	0.43
	Dmax (Gy)	19.26	20.8	0.64

## Discussion

RT is a crucial component in the management of esophageal cancer. However, it can adversely affect the heart, leading to conditions such as myocardial fibrosis, CAD, and valvular lesions. Delayed cardiac injury, particularly myocardial fibrosis, has an incidence rate ranging from 20% to 80%, resulting in increased myocardial stiffness and decreased systolic and diastolic function, which can lead to myocardial electrical disorders, compromised heart function, or even death [14]. The exact mechanisms behind radiation-induced cardiac injury remain unclear, though direct damage from radiation is a primary factor. Research indicates that patients undergoing RT are 1.62 times more likely to die from heart disease compared to those not receiving RT [15]. However, some studies have shown that conformal radiotherapy techniques like IMRT and VMAT can reduce the risk of cardiac complications [16]. Beukema et al. emphasized the need for further follow-up on cardiac function parameters to identify critical heart regions affected by radiation [11].

The approach to managing radiation-induced CAD typically aligns with treatment protocols for standard CAD. These insights prompted an investigation into the heart's arterial system following RT. While numerous studies have explored radiation-induced cardiac injury in the context of Hodgkin’s disease and breast cancer, there is a paucity of research on the same following RT for esophageal cancer, leaving risk factors inadequately understood. Thus, we conducted this retrospective dosimetric analysis to evaluate the heart and its substructures, revealing a notable benefit of the VMAT technique, particularly in reducing high-dose volumes and mean dose, due to its superior conformity. Despite intentional cardiac sparing through optimal beam arrangement, some IMRT plans still exceeded the recommended exposure limits. This issue arose primarily from the significant contribution of the anterior beam to high-dose volumes during the initial phase of treatment. Although the VMAT technique’s superior conformity did not lead to improved lung sparing regarding low-dose volume, the use of an arc significantly increased the low-dose area.

This study provides a comprehensive dosimetric comparison between VMAT and IMRT for treating carcinoma of the esophagus, focusing on critical cardiac structures, including the heart, LAD, right atrium, right ventricle, left atrium, and left ventricle. The dosimetric parameters evaluated include V5, V10, V20, V40, V50, and Dmax. A database search was conducted to gather data on heart radiation doses. Lorenzen et al. noted that an increased mean heart dose, as opposed to the dose received by the LAD coronary artery, was associated with a heightened risk of ischemic events, though they emphasized the need for further validation of these findings [17]. Recent efforts have focused on quantifying the survival rates of esophageal cancer patients based on heart radiation dose and cardiac physiological outcomes. Moreover, Frandsen et al. stressed the critical importance of minimizing cardiac dose during RT planning [15]. Several studies have identified heart V30 as a significant predictor of radiation-induced pericardial effusion, while Tward et al. suggested V40 as a potential predictive factor [18]. In this study, the V40 of the heart was 9.84% with IMRT and 8.6% with VMAT.

A study by Konski et al., involving 102 patients treated with concurrent chemoradiotherapy for locally advanced esophageal cancer, determined that the lowest significant cutoff values for V20, V30, and V40 were 70%, 65%, and 60%, respectively [9]. In this current retrospective analysis, the V20 of the heart with IMRT was 47.35% and with VMAT was 34.94%, and V40 was 9.84% with IMRT and 8.6% with VMAT. Ogino et al. discovered that heart volumes receiving doses of ≥45 Gy (V45), ≥50 Gy (V50), and ≥55 Gy (V55) were independent risk factors for symptomatic cardiac disease in patients with esophageal cancer undergoing chemoradiotherapy [19]. In our analysis, V40 was 9.84% with IMRT and 8.6% with VMAT, and V50 was 2.4% with IMRT and 3.3% with VMAT. Previous research has shown a variety of dosimetric factors linked to cardiac events or OS in patients receiving thoracic radiation. These factors include mean heart dose, heart V45, heart V50, and heart V55, whereas heart V5 did not seem to be significant [9,20-23].

For the heart, the dosimetric values indicated that VMAT results in higher V5 and V10 values compared to IMRT (85.11% vs. 93.93%), suggesting greater low-dose exposure. However, VMAT showed lower V20 (47.35% vs. 34.94%) and V40 (9.84% vs. 8.60%) values, demonstrating superior sparing at moderate doses. The V50 was slightly higher in VMAT, but the difference was minimal. For the LAD artery, VMAT demonstrated higher V5 (66.78% vs. 92.51%) and V10 (27.26% vs. 53.44%) values but significantly lower V20 (5.73% vs. 2.33%) values compared to IMRT. VMAT also achieved lower Dmax, suggesting better sparing of the LAD at higher doses. For the right atrium, VMAT resulted in slightly higher V10 (84.7% vs. 95.67%) values compared to IMRT, indicating increased low-dose exposure but better sparing at higher doses. For the right ventricle, VMAT showed higher V5 (83.87% vs. 86.97%), V10 (54.27% vs. 70.92%), and V20 (23.34% vs. 50.14%) values, suggesting increased exposure at low to moderate doses, but it achieved a lower Dmax compared to IMRT. VMAT resulted in lower V5, V10, V20, V40, and Dmin values for the left atrium compared to IMRT, indicating better sparing at all dose levels. However, VMAT had a higher V50 value. For the left ventricle, VMAT showed lower V5, V20, V40, and Dmin values but higher V10 and Dmax values compared to IMRT, indicating better sparing at low to moderate doses.

In accordance with extensive research evaluating the relationship between cardiac dosimetric factors and outcomes in esophageal cancer patients undergoing chemoradiotherapy with or without surgery, the dosimetric advantages observed with VMAT, particularly in reducing V20 and V40 doses for critical cardiac structures, could translate into significant clinical benefits [19]. Lower moderate-dose exposure is associated with a reduced risk of radiation-induced cardiac morbidity, a crucial consideration in the long-term management of esophageal cancer patients. However, the increase in low-dose exposure to VMAT necessitates careful treatment planning and patient monitoring to optimize outcomes.

This study’s retrospective nature and limited sample size are potential limitations. Future research should focus on prospective studies with larger cohorts and longer follow-ups to validate these findings. Additionally, integrating advanced imaging and functional assessments of cardiac structures during and after treatment could provide more comprehensive insights into the clinical impacts of these dosimetric differences.

## Conclusions

VMAT may be a preferred technique for reducing the risk of radiation-induced cardiac complications in esophageal cancer treatment without compromising the dose coverage to the PTV. However, the study also highlights the need for careful treatment planning, as the increased low-dose exposure to VMAT could potentially lead to other long-term effects. Further clinical follow-up studies are necessary to validate these dosimetric benefits and assess their impact on long-term cardiac outcomes in patients undergoing radiotherapy for esophageal cancer.
